# Possible harm from glucocorticoid drugs misuse in the early phase of SARS-CoV-2 infection: a narrative review of the evidence

**DOI:** 10.1007/s11739-021-02860-3

**Published:** 2021-10-31

**Authors:** Riccardo Sarzani, Francesco Spannella, Federico Giulietti, Chiara Di Pentima, Piero Giordano, Andrea Giacometti

**Affiliations:** 1 Internal Medicine and Geriatrics, Italian National Research Centre on Aging, Hospital “U. Sestilli”, IRCCS INRCA, via della Montagnola n. 81, 60127 Ancona, Italy; 2 Department of Clinical and Molecular Sciences, University “Politecnica Delle Marche”, Via Tronto 10/a, Ancona, Italy; 3 Department of Biological Sciences and Public Health, Infectious Diseases Clinic, University “Politecnica Delle Marche”, Via Tronto 10/a, Ancona, Italy

**Keywords:** SARS-CoV-2, Glucocorticoid, Innate immunity, Interferon, Cytokine, Viral infection

## Abstract

Since the publication of the RECOVERY trial, the use of glucocorticoid drugs (GC) has spread for the treatment of severe COVID-19 worldwide. However, the benefit of dexamethasone was largest in patients who received mechanical ventilation or supplemental oxygen therapy, while no benefit was found among patients without hypoxemia. In addition, a positive outcome was found in patients who received dexamethasone after several days of symptoms, while possible harm could exist if administered early. The right time interval for GC administration is still a matter of debate. Previous studies showed that an early GC use during the first phase of the disease, when viral replication peaks, may negatively affect the innate immune response through several mechanisms, such as the inhibition of pro-inflammatory and antiviral cytokine production and signaling pathway, including type I interferon, that is fundamental to counteract the virus and that was found to be impaired in several patients with life-threatening COVID-19. The GC misuse can lead to a more severe disease even in patients who do not have the established risk factors, such as obesity and cardiovascular diseases. In our focused review, we describe the role of immune response in viral infections, especially SARS-CoV-2, and discuss the potential harms of GC misuse in COVID-19.

**Supplementary Information:**

The online version contains supplementary material available at 10.1007/s11739-021-02860-3.

## Introduction

Italy is one of the countries with the highest mortality rate for coronavirus disease 2019 (COVID-19), especially in subject aged 80 + . Since April 2021, after the beginning of the extensive vaccination campaign, especially to the older population, we have seen an almost steady decrease in the number of COVID-19 patients hospitalized or deceased compared to the previous period [1]. However, also due to the spread of SARS-CoV-2 variants, the battle against the virus is not yet won [2, 3].

In October 2020, the Italian Medicines Agency (Agenzia Italiana del Farmaco—AIFA) started to support the use of glucocorticoid drugs (GC), especially dexamethasone, in COVID-19 patients who required supplemental oxygen therapy and ventilation, mainly due to the positive findings of the RECOVERY trial [4]. Since then, the use of glucocorticoids (GC) has spread for the treatment of severe COVID-19 during the so-called “third wave of COVID-19” in Italy. Although there are recommendations on how to use GC in COVID-19 patients [5], several aspects of this therapy (i.e., dosages, time interval, patient selection) are still a matter of debate, needing more clinical trials to be clarified. In this context, GC misuse could be harmful for the patients, especially in the early phase of disease. In the following narrative review, we will discuss the possible implications of GC misuse in viral infections, especially COVID-19, from an immunological and a clinical point of view.

## Methods

We searched Medline (PubMed) and Scopus to identify the published studies. The main search was run on March 2021 and updated until September 2021. We took into account observational studies (prospective or retrospective cohort, case–control or cross-sectional studies), case series and case reports, editorials, comments, clinical trials, review articles and meta-analyses published in the last 20 years. The search was limited to English language studies published in peer-reviewed journals. The keywords regarding the topics addressed (es. COVID-19, viral infection, glucocorticoids, immune system, cytokines, IL-6 antagonists) were typed in various combinations using Boolean operators (see the detailed keywords in supplemental material). Hand searches of reference lists of articles and relevant literature reviews were also used. For the preparation of this review, authors reviewed all the relevant articles found to ensure that they could address our review questions, and decided the studies to include by consensus, quality journals, articles with the most relevant data on the topic of interest and on the basis of our purpose, to make a comprehensive narrative synthesis of the available published information.

## The role of innate and adaptive immune response against SARS-CoV-2 and other viral infections

The innate immune system provides the first line of defense against viruses [6]. Upon infecting cells in the respiratory tract, the single-strand RNA (ssRNA) structure of SARS-CoV-2 is recognized by Toll-like receptor 7 (TLR7) within endosomes, which leads to the induction of pro-inflammatory cytokines and interferons (IFN). In a large part of healthy individuals, this immune response is sufficient to clear the virus and prevent the development of severe disease. TLR7 was also a pattern recognition receptor for ssRNA of both MERS-CoV and SARS-CoV. It likely plays a key role also in SARS-CoV-2 infection, given that this coronavirus contains even more ssRNA motifs that could interact with TLR7 [6]. Rare putative loss-of-function variants of X-chromosomal TLR7 were detected in young men (age < 35 years) without preexisting medical conditions affected by severe COVID-19 [7]. Loss-of-function variants in TLR7 were associated with impaired type I and II IFN responses to coronavirus, that can lead to both impaired viral clearance and increased direct cytopathic viral effects due to the higher viral load [7]. Therefore, TLR7 function, together with type I and II IFN response, is likely to be of paramount importance to avoid severe COVID-19, which is largely related to viral load and persistence, resulting in diffuse damage of lung cells and plasma protein leakage into alveolar spaces [8]. Noteworthy, patients with severe COVID-19 exhibit impaired type I and type II IFN responses and lower viral clearance in clinical studies [9, 10]. Inborn errors of type I IFN immunity with loss-of-function, involving human loci known to govern TLR3 and interferon regulatory factor 7 (IRF7), were found in life-threatening COVID-19 patients [11]. Again, autoantibodies against interferons were found in critical patients, while these ones were not found in individuals with asymptomatic or mild SARS-CoV-2 infection [12]. Clinical trials on the administration of different interferon types have been conducted to evaluate the response to symptoms and outcomes in early phases of COVID-19, albeit with inconclusive results [13]. As regards adaptive immunity, SARS-CoV-2-specific antibodies, CD4 + T-cells, and CD8 + T-cells have protective role in controlling SARS-CoV-2 infection [14]. T-cell responses have been associated with control of primary infection. Among T-cells, CD4 + cells play a key role [14–16]. Indeed, if quickly inducted, since the first days post-symptom onset, SARS-CoV-2-specific CD4 + T-cells accelerate viral clearance and their level have the strongest association with attenuated COVID-19 disease severity, compared to antibodies and CD8 + T-cells [16, 17]. Virus-specific CD4 + T-cells can differentiate into a range of helper and effector cell types: Type-1 helper T-cells, which have direct antiviral activities producing IFN-γ and related cytokines, and T follicular helper cells, which provide B-cell maturation and development of most neutralizing antibodies, as well as memory B-cells [18]. In SARS-CoV-2 infection, also the high levels of virus-specific CD8 + T-cells have been associated with better COVID-19 outcomes, producing high levels of molecules with potent cytotoxic effector functions, such as IFN-γ, granzyme B, perforin, and CD107a [15, 16, 19]. CD8 cells are the most found among infiltrating cells in pathological examinations [20]. Even if T-cell have a protective role, their dysfunctional response accounted, in part, for the severe immune injury in COVID-19 with an exacerbated inflammatory response [21]. Neutralizing antibodies develop rapidly in most SARS-CoV-2-infected people. The vast majority seroconvert within 5–15 day post-symptoms onset [22] and spike protein is the main target of SARS-CoV-2 neutralizing antibodies. The presence of IgG to spike antigens correlates with time to a negative swab result, providing the best correlate of neutralization within the first weeks from symptoms onset [23]. However, around 1–2 weeks after symptoms onset, severely ill patients show a peak in inflammation, edema, and thrombosis. This is suggested not to be a direct effect of viral infection, but instead to be strictly related to seroconversion and to overactivation of adaptive immune response [24]. Indeed, it has been shown how anti-spike IgG from patients with severe COVID-19 has an increased inflammatory potential due to a different glycosylation, particularly low fucosylation of the antibody Fc tail [25]. Right after seroconversion, anti-spike IgG titers in these patients are higher and Fc glycosylation is most aberrant [24, 26]. “Pathogenic” anti-spike IgG amplify proinflammatory responses by human alveolar macrophages, through Fcγ Receptor (FcγR) IIa and FcγRIII activation, which induces cytokines production [27]. All this evidence emphasizes the key role played by innate and adaptive immunity, and its dysregulation, in controlling SARS-CoV-2 viral load and subsequent COVID-19 severity. Moreover, it could at least in part explain the wide inter-individual variability of SARS-CoV-2 clinical picture, ranging from silent infection to deadly disease, adding to the now well-known risk factors for worse outcomes, such as advanced age, obesity and cardiovascular diseases.

## Possible harm related to the use of glucocorticoid drugs in viral infections

**Fig. 1:**
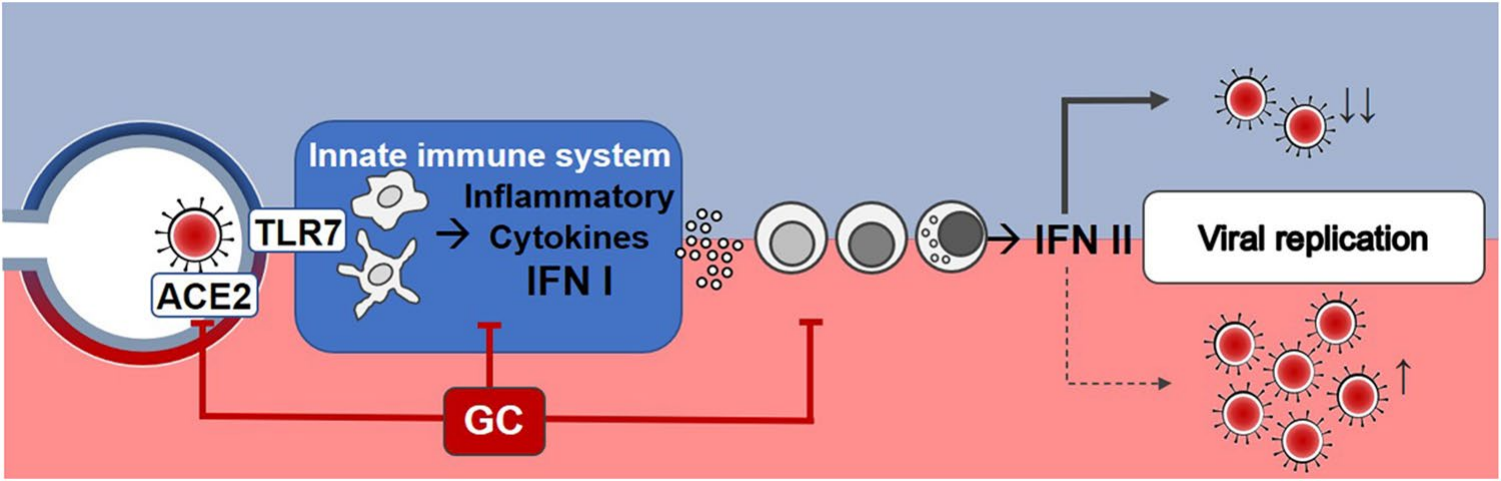
Impairment of the innate immune response against SARS-CoV-2 infection by early glucocorticoid therapy. At the top of the figure (blue part), the physiological innate immune response to viral infection (SARS-CoV-2) is summarized. Infected cells as well as macrophages and dendritic cells recognize the viral single-stranded RNA mainly through intracellular TLR7, inducing the transcription and subsequent secretion of inflammatory cytokines and type I interferon (IFN-I). They enhance antigen presentation and activate the adaptive immune system (antibody production, increased effector T-cell responses, production of type II interferon by activated T-cells and natural killer cells, etc.), counteracting viral replication. At the bottom of the figure (red part), the possible negative effects of early glucocorticoid therapy on immune response is described. Glucocorticoid therapy inhibits pro-inflammatory (IL-6 and IL-8) and antiviral (IFN-I) cytokine production and signaling pathway, decreasing the expression of the interferon-stimulated genes, suppress antigen-stimulated inflammation mediated by macrophages and dendritic cells. Glucocorticoid therapy also induces lymphopenia or can worsen a preexisting lymphopenia, hindering the T-lymphocyte immunity. Moreover, it can further downregulate membrane-bound angiotensin-converting enzyme 2 (ACE2). All these actions may contribute to viral replication and more severe lung injury. ACE2: angiotensin converting enzyme 2; TLR7: toll-like receptor 7; IFN: interferon; GC: glucocorticoid

GC may affect the immune response to viral infections through several mechanisms. Indeed, they downregulate IFN-γ production, suppress antigen-stimulated inflammation mediated by macrophages and dendritic cells, impair cytotoxic immune responses of Type-1 helper T-cells, CD8 + T-cells, and natural killer (NK) cells [28]. GC inhibit pro-inflammatory (IL-6 and IL-8) and antiviral (IFN-1) cytokine production and signaling pathway, decreasing the expression of the IFN-stimulated genes [29, 30] (Fig. 1). These immune inhibitory effects, also depending on dosages, potency, and duration of treatment, may increase the susceptibility and severity of several infections. Therefore, if GC are given early in the course of a viral infection (e.g. within the first week of symptom onset when the innate response is mounting), they are likely to interfere and reduce both the efficacy of IFN production and IFN-mediated reduction of viral spread, enhancing viral replication [28, 31]. In addition, for these reasons, the clinical efficacy and safety of GC use in different viral pneumonia remains largely uncertain because of a lack of randomized trials and inconclusive observational studies. Although some reports found apparent beneficial effects of GC [32], others showed harmful effects, such as a reduced clearance of viral RNA in both severe acute respiratory syndrome (SARS) [33] and Middle East respiratory syndrome (MERS) outbreaks [34, 35]. Indeed, both Lee N. et al. and Arabi Y.M. et al. showed how the “early” initiation of GC therapy (within the first week of illness) resulted also in increased length of hospital stay and increased need for invasive ventilation in SARS and MERS, respectively [33, 34]. A recent meta-analysis summarized how GC in these infections delay virus clearing and extend the duration of hospitalization on average almost 10 days, without significant improvement in terms of mechanical ventilation use and risk of death [36]. As well as in MERS and SARS infections, a net benefit of GC has not been demonstrated even in other viral infections with a possible worse prognosis [37]. Previous studies found an association between GC therapy and poor clinical outcomes in patients affected by influenza [38]. Recent meta-analyses have shown that CG may almost twofold increase the risk of death in influenza pneumonia, extend the duration of intensive care unit stay by several days and increase the rate of secondary bacterial or fungal infection up to more than 3 times [39–41]. Indeed, the Infectious Diseases Society of America recommends against GC adjunctive therapy in patients with influenza-associated pneumonia unless clinically indicated for other reasons [42]. Clear benefits of GC treatment have not been proven even in the presence of respiratory infections by respiratory syncytial virus (RSV) in both children and adults, in which, although it has not shown to significantly affect virus shedding and immune response, it has not shown a clear improvement on clinical outcome, such as hospitalizations, either [43–45].

## Possible harm related to the use of glucocorticoid drugs in the early phase of SARS-CoV-2 infection: from pathophysiology to the clinical evidence

Since the beginning of the SARS-CoV-2 pandemic, several authors have discouraged the use of GC in COVID-19, based on previous evidence (increased mortality and risk of secondary infection in influenza impaired clearance of SARS-CoV and MERS-CoV and increased complications) and conflicting results of the first small clinical studies [37, 46]. Both high dosage of cumulative GC (≥ 200 mg of methylprednisolone-equivalent dose) and early initiation of GC therapy are risk factors for delayed viral clearance and may prolong viral shedding also in SARS-CoV-2 infection, although the major predictors of delayed SARS-CoV-2 clearance in moderate/severe COVID-19 were found to be an older age and a more severe disease [47–49], while low-dose corticosteroids probably do not have a significant impact on duration of SARS-CoV-2 viral shedding [50]. Lymphopenia might be a critical factor associated with disease severity and mortality. It is a typical characteristic of severe COVID-19, tending to persist even beyond 3 weeks after illness onset [51]. CD8 are usually present in large numbers in cellular infiltrates in the normal immune response during COVID-19 [20] and a modest lymphocytic response with small-size lymphocyte clusters was found in lung tissue from patients who died for COVID-19 [52]. GC induce lymphopenia or can worsen a preexisting lymphopenia, hindering the T-lymphocyte immunity. Early GC use is associated with lower number of CD3 + T-cells, CD8 + T-cells, and NK-cells in patients with COVID-19 pneumonia [53]. Lower levels of both CD8 T-cells and myeloid dendritic cells, together with reduced IFN response, were found in critically ill patients, who required high-flow oxygen therapy and mechanical ventilation, compared to non-critically ill patients who received low-flow oxygen therapy [54]. These aspects could at least partially explain the results of some early negative studies on GC. Early GC administration increased both the risk of mortality or mechanical ventilation usage in hospitalized patients with initial hs-CRP of less than 10 mg/dL, treated with GC within 48 h of admission [55]. Other clinical observational studies found that early initiation of GC (≤ 3 days after intensive care unit admission) was associated with an increased 90-day mortality also in critically ill patients with COVID-19 [56].

Furthermore, GC predispose to secondary bacterial and invasive fungal infections also in COVID-19 patients, as well as in influenza [57]. In cohort studies, a high incidence of bacterial superinfections and pulmonary aspergillosis were found among COVID-19 patients receiving mechanical ventilation during GC treatment [58, 59]. GC therapy is an independent risk factor for superimposed bacterial infections, especially gram-negative organisms [60]. Their incidence reported in the studies varies greatly according to studied population, setting and definition used [61]. In a cross-sectional study on 399 hospitalized COVID-19 patients, nearly half (49.6%) had a bacterial superinfection, mostly by Klebsiella and Staphylococcus Aureus, with an almost threefold increased risk for patients treated with GC [62]. In a case series by Wang et al., a significant higher incidence of pulmonary aspergillosis (9.5% vs 4.8%) was found in COVID-19 patients taking GC compared to those not taking GC [63]. Bacterial superinfections mostly consist of bloodstream infections [64] and hospital-acquired or ventilator-associated pneumonia [60, 65]. Not all studies have found this increased risk of superinfection with GC therapy [66, 67]. In the CAPE COVID Trial [68], GC treatment was not associated with an increase in the rate of secondary infections in critically ill COVID-19 patients, likely due to the low dosage of hydrocortisone used and, therefore, to its lower immunosuppressive effect.

Impaired glucose metabolism and diabetes mellitus are well-known risk factors for poor prognosis in COVID-19 and GC may worsen hyperglycemia and decompensate a known diabetes mellitus [69, 70], although not all studies are in agreement [59]. Zhang et al. reported hyperglycemia in 60% receiving corticosteroids vs 46% not receiving corticosteroids [71]. Both patients with diabetes and patients with secondary hyperglycemia showed higher inflammatory indices and longer hospital stays compared to controls [71]. Previous studies report how a new finding of hyperglycemia during COVID-19, due to the inflammatory state compounded by the GC therapy [72], is likely to be more strongly associated with a worse outcome than even pre-existing diabetes [73]. Therefore, careful hospital glucose management should be implemented in hospitalized COVID-19 patients receiving GC.

Less evidence in COVID-19 patients treated with GC is available regarding other adverse effects, such as avascular necrosis and osteoporosis. Only few case series reported avascular necrosis in the post-COVID-19 [74]. However, attention should be paid by physicians to this possible complication, especially if high and long-term GC doses have been used [75], given the close relationship previously found between osteonecrosis incidence and GC therapy in SARS patients [76] and that the same vascular damage by SARS-CoV-2 could predispose to necrosis [77–79]. Same considerations could be made for the risk of osteoporosis given that both single boluses of high-dose cortisone and prolonged treatments with low-dose steroids can suppress both bone formation and reabsorption, with a net loss of bone mineral mass [80].

Steroid hormones regulate multiple components of the renin–angiotensin system (RAS) [81]. Membrane-bound angiotensin-converting enzyme 2 (ACE2) is the cellular receptor for SARS-CoV-2, whose binding leads to its downregulation through its internalization and probably ACE2 shedding. ACE2 plays a very important role in lung protection, through the synthesis of angiotensin-7, exerting multiple beneficial effects (such as vasodilation, anti-hypertrophic, anti-oxidant, anti-inflammatory and anti-fibrotic actions) [8]. GC further downregulate ACE2 (Fig. 1) and recent studies found that its expression can be unregulated by INF itself [82]. It is important to note that the downregulation of membrane-bound ACE2 has a pivotal role in lung injury in COVID-19 [83]. Therefore, GC may affect COVID-19 outcome also by interfering with RAS balance.

## “Cytokine Storm” and clinical evidence supporting the use of glucocorticoid drugs in COVID-19

The “cytokine storm” is a term now widely used to describe what may happen in a more advanced phase of COVID-19 that can lead to worse outcomes. However, several criticisms undermine this definition [84]. Cytokines play a key role in coordinating antimicrobial effector cells during both innate and adaptive immune responses against invasive pathogens. Therefore, a substantial increase in several cytokine levels, from IFN-γ to interleukins and chemokines, is part of the normal response to both viral and bacterial infections. There is no clear definition of "cytokine storm" associated with viral or other infections and there is no cytokine threshold (i.e., IL-1 or IL-6) known to define the presence of a "cytokine storm". Moreover, there are difficulties in the assays of cytokines both for their short half-life and for variability assays. All these factors make hard to define the limit between a physiologic inflammatory response and a “cytokine storm”, in which the immune response and its attempt to clear the pathogen can lead to a dysregulated cytokine production and inflammatory response causing cell death, coagulopathy and multiorgan dysfunction [84]. The hypothesis of favorable effects of GC and other immunomodulators, currently recommended in COVID-19 management, derives precisely from their ability to modulate cytokine levels and, therefore, limit the inflammation-mediated lung injury.

IL-6 level is a well-known lab parameter associated with worse prognosis in COVID-19 [85]. The first conflicting results of randomized controlled trials (RCTs) on anti-IL-6 monoclonal antibodies in COVID-19 [86–90] did not show a significant mortality benefit for treatment with tocilizumab in moderately ill hospitalized patients, while a decreased risk of mechanical ventilation has been found [91]. On the other side, tocilizumab may be more effective in patients with severe COVID-19 and high levels of systemic inflammation indices. Indeed, subsequently, the REMAP-CAP trial reported a benefit of IL-6 receptor antagonist in severely ill patients requiring organ support, with improvement in survival, reducing mortality by 8.5%, and improvement in length of stay, with earlier discharge from the intensive care unit [92]. Lately, the RECOVERY trial [93] confirmed that tocilizumab improved survival and reduced invasive mechanical ventilation need in hospitalized COVID-19 patients with hypoxia and systemic inflammation. Moreover, tocilizumab improved the chances of discharge from hospital by 28 days. Recently, a meta-analysis summarized 27 randomized trials of IL-6 receptor antagonists [94]. Although further studies are needed, the authors found significant mortality benefit limited to tocilizumab when co-administered with GC and among patients who received respiratory support with oxygen by nasal cannula, face mask, high-flow nasal oxygen or noninvasive ventilation [94, 95]. Indeed, the coadministration of GC with immunomodulatory treatments, such as IL-6 receptor antagonists but also JAK inhibitors [96] may provide additive benefits in reducing overall mortality in hospitalized patients with COVID-19 receiving supplemental oxygen therapy or ventilation [97].

The positive data coming from RCTs favoring the use of GC are mainly related to dexamethasone in severe hospitalized COVID-19 patients requiring respiratory support or supplemental oxygen, in which it was found to improve the outcome (need for invasive mechanical ventilation, ventilator-free days and death) [4, 98–100]. It is interesting to note that, in the RECOVERY trial, the median number of days from symptoms onset were 9 days in the “oxygen-only group” and 13 days in the “invasive mechanical ventilation group”, respectively [4], therefore, beyond what can be defined an early stage of the disease, but a more advanced phase, in which viral replication was likely declined in most patients. Moreover, dexamethasone 6 mg was associated with a reduction in mortality only among those who had symptoms for more than 7 days, while a trend for possible worse outcome was found in patients with milder and earlier disease [4]. In agreement with these findings, several retrospective observational studies found that patients affected by SARS-CoV-2 pneumonia / acute respiratory distress syndrome (ARDS) who had been treated with GC therapy started with a median time from symptom onset equal to or greater than 8–10 days, had lower in-hospital mortality than controls [101–103]. Inversely, starting GC before 7 days of symptom onset was not associated with lower mortality in a population of 571 hospitalized COVID-19 patients analyzed by Bahl et al. [103]. All these data suggest that GC should only be given in severe cases and several days after the onset of the disease/symptoms. However, the right time interval for GC administration is still a subject of debate and further studies are needed. In addition to the time from symptom onset, the response to GC treatment is likely related also to CRP levels and degree of lymphopenia [55, 67, 104]. Moreover, there are still doubts on the correct duration of the GC therapy (up to 10 days in the RECOVERY trial [4]), given that prolonged corticosteroid treatment may not be harmless, in terms of interference with coagulation and metabolic pathways, and long-term symptoms [105]. Finally, there are other concerns on the generalization of the RECOVERY trial data, given the several limitations of the study, regarding inclusion criteria, analyzed data and phenotyping [105]. A recent meta-analysis attempted to summarize the evidences available on the use of GC in COVID-19, highlighting the heterogeneity among the studies. Favorable effects were found in severely ill COVID-19 patients, while no benefit or even harm was found in the overall analysis [50]. Another aspect that emerges from this meta-analysis is the scarce evidence regarding the right dosage, if low-dose regimens, which seem to have no significant impact on viral shedding, or high-dose regimens are needed to obtain a clinical benefits [50]. Precisely, the low dosage/underdosing and the missing therapeutic window could perhaps explain both the lack of effect reported in some studies and the high heterogeneity in findings between them. In fact, the discrepancies in the results are more evident among the studies that used low dosages of GC [50, 98]. All this debate reflects how the role of GC therapy in COVID-19 still needs to be fully elucidated in clinical practice, particularly regarding the careful selection of the patients who could benefit from it.

## Conclusion

COVID-19 likely consists of two main phases: the first one, in which viral replication peaks and a direct viral damage (mainly, but not only) to the lung is found, and the second one, in which a dysregulated inflammatory response could lead to systemic effects and worse prognosis [106]. All the evidence supporting GC administration was found in the second phase, when the dysregulated inflammatory response can contribute to organ damage. On the contrary, GC use could be harmful in the earlier phase, when the innate immunity response is fighting against viral replication. Therefore, there is neither evidence nor a rationale for the use of GC in the first phase of SARS-CoV-2 infection, especially in asymptomatic non-hospitalized patients. On the contrary, there are enough data to suggest that GC use in this phase may be harmful by promoting SARS-CoV-2 replication for longer time, increasing viral load, dysregulating the normal innate immunity with consequent possible worse lung damage. All this could contribute to increase the rates of both hospitalizations and the number of severe cases requiring respiratory support, even in younger subjects without comorbidities. In this context, the GC misuse in the first phase of SARS-CoV-2 infection should be discouraged and the indication should be restricted to selected hospitalized cases. Therefore, it is important to stress that to date, GC are recommended only in patients with severe or critical COVID-19 who require oxygen supplementation, both conventional oxygen therapy and mechanical ventilation. On the contrary, GC may be harmful and are not recommended in non-severe COVID-19, namely, outpatients who do not require additional oxygen therapy. These evidences have now been widely accepted by both international (World Health Organization [107]) and local (Italian National Institute of Health [108] and Italian Society of General Medicine [109]) recommendations on home management of COVID-19, which discourage early use of GC as “home therapy”.

## Supplementary Information

Below is the link to the electronic supplementary material.

Supplementary file1 (DOCX 15 KB)
