# Supplementary material for: Possible harm from glucocorticoid drugs misuse in the early phase of SARS-CoV-2 infection: a narrative review of the evidence
Source: Intern Emerg Med. 2021 Oct 31;17(2):329–38. doi: 10.1007/s11739-021-02860-3 (PMC8557262; doi:10.1007/s11739-021-02860-3)
Supplement: Supplementary file 1 — Supplementary file1 (DOCX 15 KB) [file 11739_2021_2860_MOESM1_ESM.docx]

Search strategy used for PubMed (Medline) and Scopus.

Keywords:

1. ACE2
2. Adaptive immunity
3. Adverse effects
4. Antibodies
5. Avascular necrosis
6. B-cells
7. Corticosteroid
8. Corticosteroids
9. COVID-19
10. COVID-19 vaccine
11. Cytokines
12. Dexamethasone
13. Diabetes mellitus
14. Dosage
15. Duration
16. Early initiation
17. Early use
18. Efficacy
19. Glucocorticoid
20. Guidelines
21. Harms
22. Hospitalization
23. Hydrocortisone
24. Hyperglycemia
25. IL-6
26. IL-6 antagonist
27. Immune response
28. Inflammation
29. Influenza
30. Innate immunity
31. Interferon
32. Lymphocytes
33. Lymphopenia
34. Mechanical ventilation
35. MERS-CoV
36. Meta-analysis
37. Methylprednisolone
38. Mortality
39. Osteoporosis
40. Outcome
41. Prognosis
42. Recommendations
43. RECOVERY trial
44. REMAP-CAP trial
45. Renin-angiotensin system
46. Respiratory infections
47. Safety
48. SARS-CoV
49. SARS-CoV-2
50. Secondary infections
51. Severe COVID-19
52. Severity
53. Side effects
54. Steroid
55. Steroids
56. Superinfections
57. Symptoms
58. T-cells
59. Timing
60. Tocilizumab
61. Viral clearance
62. Viral infections
63. Viral pneumonia
64. Viral shedding
65. Viral spread
